# Effect of elevated CEA levels on the outcome of colorectal cancer patients with different histopathologic types: A SEER population-based study

**DOI:** 10.17305/bb.2024.11265

**Published:** 2024-10-16

**Authors:** Siqi Sheng, Xiaoming Bai, Yiting Wang, Haimei Feng, Jie Chen, Yitian Chen, Mengxi Huang, Zengjie Lei, Xiaoyuan Chu

**Affiliations:** 1 Department of Medical Oncology, Affiliated Jinling Hospital, Medical School, Nanjing University, Nanjing, Jiangsu, China; 2 Jinling Clinical Medical College, Nanjing University of Chinese Medicine, Nanjing, Jiangsu, China; 3 Department of Oncology, The Second Hospital of Nanjing, Nanjing University of Chinese Medicine, Nanjing, Jiangsu, China; 4 Department of Medical Oncology, Jinling Hospital, Nanjing Medical University, Nanjing, Jiangsu, China; 5 Department of Medical Oncology, Jinling Hospital, The First School of Clinical Medicine, Southern Medical University, Nanjing, Jiangsu, China

**Keywords:** Colorectal cancer, carcinoembryonic antigen, histopathological type, prognosis, metastasis

## Abstract

Limited and contradictory evidence has been reported regarding the prognostic effects of carcinoembryonic antigen (CEA) on the prognosis and metastasis of classical adenocarcinoma (CA), mucinous adenocarcinoma (MA), and signet-ring cell carcinoma (SRCC) in colorectal cancer (CRC) patients. We investigated the associations between histological subtypes and preoperative serum CEA levels in determining the oncologic outcomes of CRC patients. A total of 47,692 patients with clearly diagnosed CRC were selected from the Surveillance, Epidemiology, and End Results (SEER) database and divided into two cohorts based on serum CEA levels: CEA-normal (C0) and CEA-elevated (C1). Chi-square analysis revealed a correlation between CEA levels and histological classification. We then included a newly defined interaction variable (H&CEA) in the Cox regression analysis, which demonstrated that this variable could serve as an independent prognostic factor (*P* < 0.001). CA, in the context of elevated serum CEA levels, differed from the other two histopathological types, showing unexpectedly higher risks for both overall survival (OS) (HR ═ 1.70, 95% CI ═ 1.65–1.75, *P* < 0.001) and cancer-specific survival (CSS) (HR ═ 1.78, 95% CI ═ 1.72–1.85, *P* < 0.001). Furthermore, elevated CEA levels significantly increased the proportion of liver metastases in the CA group (25.43% vs 3.95%, *P* < 0.001). The interaction variable H&CEA can be used as an independent prognostic factor for CRC and should be considered in the diagnosis of CRC and the development of personalized treatment plans. Additionally, in the context of elevated CEA levels, CA is associated with poor prognosis and increased liver metastases. This CRC subgroup warrants special clinical attention from oncologists.

## Introduction

According to global cancer statistics from 2023, colorectal cancer (CRC) is the third most prevalent malignant tumor and the second leading cause of cancer-related death worldwide [1]. Its high malignancy, rapid progression, and significant morbidity and mortality now pose major public health challenges. Radical surgery remains the primary treatment for CRC. In recent years, improvements in overall survival (OS) and prognosis may be attributed to the development of personalized treatment plans based on advancements in understanding CRC pathophysiology [2]. However, postoperative tumor recurrence and metastasis continue to be significant issues.

Histological analysis of postoperative specimens reveals that CRC comprises various subtypes, with adenocarcinoma being the most common [3]. Based on the mucinous content of the primary tumor, adenocarcinoma can be classified into three well-studied subtypes: classical adenocarcinoma (CA), mucinous adenocarcinoma (MA), and signet-ring cell carcinoma (SRCC) [4]. Previous studies have shown that the CRC histological subtype has prognostic significance. Patients with MA exhibit poorer prognoses compared to those with CA [5], and the presence of SRCC is an independent indicator of poor prognosis in CRC patients [6]. Although CA is often associated with a more favorable prognosis, its clinical outcomes can vary considerably. Given the high incidence of CA, further studies are warranted to investigate the prognostic factors specific to this histological subtype.

In 1965, carcinoembryonic antigen (CEA) was identified as a member of the immunoglobulin superfamily, with a molecular weight of 180–200 kDa [7]. CEA, secreted by various solid tumors, is a crucial and irreplaceable prognostic biomarker in CRC. About 90% of CRC tumors express CEA, which has been reported to accelerate tumor progression, promote colon cancer cell adhesion to metastatic sites, and correlate with poor long-term survival outcomes [8, 9].

To our knowledge, this is the first systematic retrospective study investigating the association between histological classification and preoperative serum CEA levels (C0 and C1) in predicting prognosis and metastasis in CRC patients, using a large sample from the Surveillance, Epidemiology, and End Results (SEER) database. Understanding these patterns could provide valuable insights into CRC epidemiology and help guide clinical decisions regarding surveillance and adjuvant treatment after CRC.

## Materials and methods

### Patient selection

Data for all patients in the present study were retrieved from the SEER database, which contains information from population-based cancer registries on patient demographics, cancer incidence, treatment, and outcomes (https://seer.cancer.gov). Initially, the case-listing session of the SEER^*^Stat software (SEER*Stat 8.3.9) was used to summarize all patient-related information. Patients diagnosed with CRC between January 1, 2004, and December 31, 2015, were identified from the SEER database. This time frame was chosen because pretreatment serum CEA data became available starting in 2004, and we aimed to have at least three years of follow-up data. Informed consent was not required from patients because the SEER database is publicly accessible. The following criteria were used for patients with CRC:

(1) Patients aged between 20 and 79 diagnosed with CRC with malignant behavior between 2004 and 2015.
(2) Excluded patients diagnosed only by death certificates or autopsy.
(3) Excluded patients without pathologically confirmed diagnoses.
(4) Patients with histopathology of CA, MA, or SRCC.
(5) Patients with detailed information, including site, grade, T stage, N stage, M stage, age, sex, race, survival mouth, and Preoperative CEA level.

**Figure 1.**
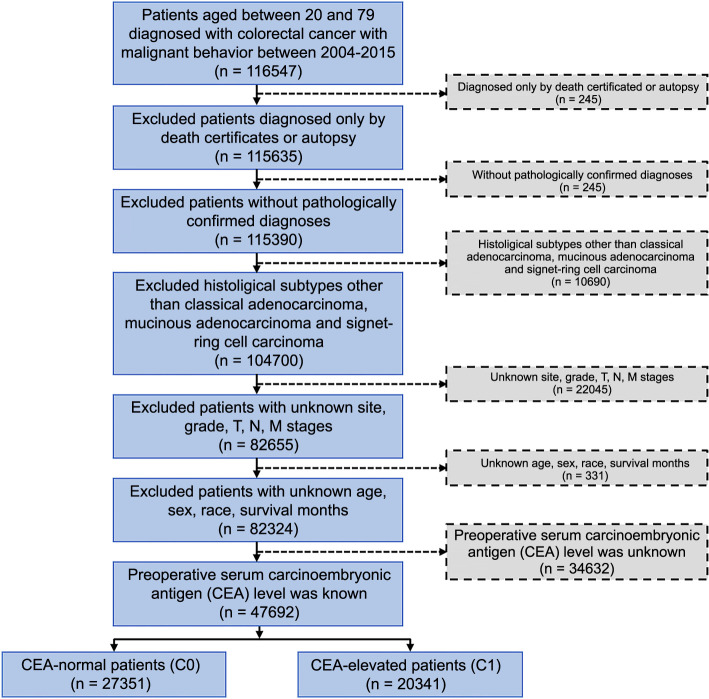
**The flow diagram of eligible patients selected from the SEER database.** CEA: Carcinoembryonic antigen.

A flowchart of the selection criteria for patients is presented in Figure 1.

### Clinicopathological factors

The following clinicopathological variables were extracted for analysis: race, sex, age, pathology grade, historical stage, T stage, N stage, M stage, primary tumor site, histopathological types, pretreatment serum CEA levels, OS, cancer-specific survival (CSS), and metastatic tumor site. According to the International Classification of Diseases in Oncology (ICDO-3), tumors with codes 8140–8147, 8210–8213, 8220–8221, 8255, 8260–8263, and 8310–8323 were classified as CA, while those with codes 8480–8481 were categorized as AC, and those with code 8490 as SRCC. Patients diagnosed with other histological subtypes were excluded. In addition, we grouped CEA levels as “positive/elevated” (C1) and “negative/normal” (C0). The cutoff value for the CEA level was set at 5 ng/mL. Consequently, all included CRC patients were divided into two cohorts based on their CEA levels: C0 and C1. The primary outcomes were OS, CSS, and metastasis status. CSS was defined as death attributable to CRC, while OS included CSS as well as death from other causes.

### Ethical statement

The data used in this study were anonymized and collected from a publicly available database; therefore, no ethical approval was required.

### Statistical analysis

In this study, Pearson’s chi-square test was used to compare the clinicopathological variables between patients with normal and elevated preoperative serum CEA levels in the SEER database. Univariate and multivariate Cox regression analyses were then performed to identify independent prognostic factors for CRC. Additionally, we introduced a new variable combining histological subtype and CEA level to determine if there was a significant interaction effect between these two factors on OS or CSS. The differences in OS and CSS between the six H&CEA groups were visualized using survival curves generated by the Kaplan–Meier method, and hazard ratios (HRs) were displayed in a forest plot. Metastasis status was also analyzed as a study endpoint. Similarly, chi-square tests were performed to evaluate the distribution of metastatic sites. Finally, logistic regression analysis was conducted to compare the occurrence of single-site metastasis between the normal- and elevated-CEA groups based on histopathological type. All statistical analyses were performed using R software (version 4.0.1, www.r-project.org), with the main packages used being ggplot2, MatchIt, survival, rms, and survminer. Additional graphics were created using GraphPad Prism 8 (GraphPad Software, CA, USA). Results were considered statistically significant when the two-sided *P* value was less than 0.001 for all analyses.

## Results

### Basic clinical characteristics of CRC patients

As shown in Figure 1, a total of 47,692 patients were enrolled in our study. All patients were divided into two groups according to preoperative CEA levels, including 27,351 (57.3%) patients in the CEA-normal (C0) group and 20,341 (42.7%) patients in the CEA-elevated (C1) group. The demographic and clinical characteristics of the patients in both groups were analyzed in Table 1. Significant differences were observed in race (*P* < 0.001), sex (*P* ═ 0.002), grade (*P* < 0.001), stage (*P* < 0.001), T stage (*P* < 0.001), N stage (*P* < 0.001), M stage (*P* < 0.001), site (*P* < 0.001), and histological classification (*P* < 0.001) between the C0 and C1 groups, but no significant difference was found in age (*P* ═ 0.648). Compared with the patients in the C0 group, those in the C1 group were more likely to be Black (14.17% vs 9.42%) and female (45.95% vs 44.52%), to have poorly or undifferentiated tumors (22.25% vs 17.6%), to have advanced tumors (35.26% vs 7.48%), to have a larger tumor size, defined as T3 (59.90% vs 53.65%) or T4 (23.91% vs 9.96%), to have a higher N stage (25.50% vs 13.07%), to have metastatic tumors (33.61% vs 6.73%), and to have tumors located in the left-side colon (38.09% vs 37.06%) or rectum (22.11% vs 20.91%). Additionally, the CEA-elevated group was more likely to have MA (8.55% vs 6.86%) or signet ring cell carcinoma (1.3% vs 0.77%) than the CEA-normal group.

**Table 1:** Demographics and clinicopathological characteristics of patients with C0 or C1

**Variables**	**CEA-normal (C0)** ***n* ═ 27,351** ***n*(%)**	**CEA-elevated (C1)** ***n* ═ 20,341** ***n*(%)**	***P***
*Race*			
White	21478 (78.53)	14677 (72.15)	<0.001
Black	2577 (9.42)	2882 (14.17)	
Other	3296 (12.05)	2782 (13.68)	
*Sex*			
Male	15174 (55.48)	10994 (54.05)	0.002
Female	12177 (44.52)	9347 (45.95)	
*Age*			
<=65	15445 (56.47)	11530 (56.68)	0.648
>65	11906 (43.53)	8811 (43.32)	
*Grade*			
I	2174 (7.95)	1158 (5.69)	<0.001
II	20364 (74.45)	14658 (72.06)	
III	4214 (15.41)	3986 (19.6)	
IV	599 (2.19)	539 (2.65)	
*Stage*			
Distant	2046 (7.48)	7173 (35.26)	<0.001
Localized	12231 (44.72)	4172 (20.51)	
Regional	13074 (47.8)	8996 (44.23)	
*T*			
T1	4958 (18.13)	1601 (7.87)	<0.001
T2	4995 (18.26)	1693 (8.32)	
T3	14674 (53.65)	12184 (59.9)	
T4	2724 (9.96)	4863 (23.91)	
*N*			
N0	16546 (60.5)	8454 (41.56)	<0.001
N1	7230 (26.43)	6701 (32.94)	
N2	3575 (13.07)	5186 (25.5)	
*M*			
M0	25510 (93.27)	13505 (66.39)	<0.001
M1	1841 (6.73)	6836 (33.61)	
*Site*			
Right	11496 (42.03)	8096 (39.8)	<0.001
Left	10136 (37.06)	7747 (38.09)	
Rectal	5719 (20.91)	4498 (22.11)	
*Histopathologic type*			
CA	25266 (92.38)	18338 (90.15)	<0.001
MA	1875(6.86)	1739(8.55)	
SRCC	210 (0.77)	264 (1.3)	

CEA: Carcinoembryonic antigen; C0: CEA-normal; C1: CEA-elevated; CA: Classical adenocarcinoma; MA: Mucinous adenocarcinoma; SRCC: Signet-ring cell carcinoma.

### Improved risk of CA compared with other histopathological types in the context of serum CEA elevation

Through univariate and multivariate Cox regression analyses, we explored the prognostic factors of CRC in the entire population from the SEER database (Table S1). The factors included in the analyses were race, sex, age, grade, stage, T stage, N stage, M stage, CEA level, tumor site, and histopathologic type. Univariate analyses revealed that all of these factors were risk factors for OS in CRC patients, consistent with the results of the multivariate logistic regression analysis. Given that the proportion of adenocarcinoma patients with elevated CEA levels was lower than that of patients with normal CEA levels, we next investigated whether CEA was associated with different histopathologic types of CRC. Thus, univariate and multivariate Cox regression analyses for OS across the three pathology subgroups were carried out (Table 2). We found that, after adjusting for potential confounders via multivariate logistic regression analysis, several factors, such as sex and tumor grade, were risk factors for OS only in patients with adenocarcinoma (*P* < 0.001) but had no significant effect in patients with MC or SRCC. However, elevated CEA levels were an independent risk factor for OS across all three subgroups.

**Table 2:** Univariate and multivariate analyses for CRC patients in different histopathologic types

**Classical adenocarcinoma (CA)**							**Mucinous adenocarcinoma (MA)**							**Signet-ring cell carcinoma (SRCC)**						
**Variables**	**Univariate analysis of OS**			**Multivariate analysis of OS**			**Variables**	**Univariate analysis of OS**			**Multivariate analysis of OS**			**Variables**	**Univariate analysis of OS**			**Multivariate analysis of OS**		
	**Hazard** **ratio**	**95% CI**	***P***	**Hazard**> **ratio**	**95% CI**	***P***		**Hazard**> **ratio**	**95% CI**	***P***	**Hazard** **ratio**	**95% CI**	***P***		**Hazard** **ratio**	**95% CI**	***P***	**Hazard** **ratio**	**95% CI**	***P***
*Race*							*Race*							*Race*						
White	Ref			Ref			White	Ref			Ref			White	Ref					
Black	1.37	1.32–1.43	<0.001	1.34	1.28–1.40	<0.001	Black	1.20	1.05–1.37	0.008	1.31	1.14–1.50	<0.001	Black	1.24	0.88–1.75	0.222			
Other	0.90	0.86–0.94	<0.001	0.95	0.91–0.99	0.018	Other	0.92	0.78-1.09	0.330	0.89	0.75–1.05	0.157	Other	0.93	0.64–1.34	0.697			
*Sex*							*Sex*							*Sex*						
Male	Ref			Ref			Male	Ref						Male	Ref					
Female	0.86	0.84–0.89	<0.001	0.82	0.79–0.84	<0.001	Female	0.99	0.90–1.08	0.754				Female	0.85	0.69–1.05	0.138			
*Age*							*Age*							*Age*						
<=65	Ref			Ref			<=65	Ref			Ref			<=65	Ref					
>65	1.61	1.56–1.65	<0.001	1.97	1.91–2.02	<0.001	>65	1.38	1.26–1.51	<0.001	1.63	1.48–1.79	<0.001	>65	1.23	0.99–1.52	0.061			
*Grade*							*Grade*							*Grade*						
I	Ref			Ref			I	Ref			Ref			I	Ref					
II	1.23	1.16–1.30	<0.001	1.00	0.94–1.06	0.927	II	0.94	0.80–1.10	0.417	0.88	0.75–1.04	0.124	II	0.82	0.19–3.6	0.795			
III	1.94	1.81–2.07	<0.001	1.26	1.18–1.35	<0.001	III	1.36	1.14–1.63	0.001	1.07	0.89–1.28	0.478	III	1.27	0.32–5.11	0.736			
IV	2.00	1.8–2.22	<0.001	1.30	1.17–1.45	<0.001	IV	1.48	1.14–1.92	0.003	1.02	0.78–1.33	0.882	IV	1.08	0.26–4.44	0.916			
*Stage*							*Stage*							*Stage*						
Distant	Ref			Ref			Distant	Ref			Ref			Distant	Ref			Ref		
Localized	0.14	0.14–0.15	<0.001	0.59	0.52–0.67	<0.001	Localized	0.17	0.15–0.20	<0.001	0.87	0.60–1.28	0.482	Localized	0.09	0.05–0.16	<0.001	0.29	0.10–0.86	0.025
Regional	0.22	0.21–0.23	<0.001	0.71	0.62–0.80	<0.001	Regional	0.26	0.23–0.28	<0.001	0.91	0.64–1.29	0.604	Regional	0.25	0.20–0.32	<0.001	0.7	0.28–1.75	0.449
*T*							*T*							*T*						
T1	Ref			Ref			T1	Ref			Ref			T1	Ref			Ref		
T2	0.86	0.81–0.92	<0.001	0.83	0.78–0.88	<0.001	T2	0.63	0.48–0.84	0.001	0.65	0.49–0.85	0.002	T2	0.55	0.26–1.18	0.125	0.46	0.21–1.00	0.049
T3	1.52	1.45–1.59	<0.001	0.96	0.91–1.01	0.133	T3	0.98	0.78–1.24	0.874	0.72	0.56–0.91	0.007	T3	1.06	0.64–1.75	0.829	0.51	0.29–0.89	0.017
T4	3.28	3.11–3.45	<0.001	1.38	1.3–1.460	<0.001	T4	1.97	1.55–2.50	<0.001	1.04	0.80–1.35	0.761	T4	2.39	1.45–3.94	0.001	0.75	0.43–1.32	0.318
*N*							*N*							*N*						
N0	Ref			Ref			N0	Ref			Ref			N0	Ref			Ref		
N1	1.54	1.49–1.59	<0.01	1.07	1.03–1.11	0.001	N1	1.53	1.37–1.71	<0.001	1.29	1.13–1.47	<0.001	N1	1.3	0.92–1.86	0.140	0.99	0.67–1.46	0.961
N2	2.77	2.67–2.87	<0.01	1.45	1.39–1.52	<0.001	N2	2.90	2.60–3.23	<0.001	1.86	1.62–2.13	<0.001	N2	2.67	2.00–3.58	<0.001	1.62	1.16–2.27	0.004
*M*							*M*							*M*						
M0	Ref			Ref			M0	Ref			Ref			M0	Ref			Ref		
M1	5.75	5.58–5.93	<0.01	2.79	2.46–3.16	<0.001	M1	4.95	4.47–5.48	<0.001	3.17	2.23–4.50	<0.001	M1	4.58	3.64–5.77	<0.001	2.37	0.96–5.83	0.060
*CEA*							*CEA*							*CEA*						
C0	Ref			Ref			C0	Ref			Ref			C0	Ref			Ref		
C1	2.64	2.57–2.72	<0.01	1.70	1.65–1.76	<0.001	C1	1.88	1.71–2.06	<0.001	1.37	1.24–1.51	<0.001	C1	1.44	1.16–1.79	0.001	1.42	1.14–1.77	0.002
*Site*							*Site*							*Site*						
Right	Ref			Ref			Right	Ref			Ref			Right	Ref					
Left	0.88	0.85–0.90	<0.01	0.88	0.86–0.91	<0.001	Left	1.13	1.02–1.26	0.019	1.01	0.90–1.11	0.982	Left	0.88	0.68–1.15	0.352			
Rectal	0.90	0.87–0.94	<0.01	1.06	1.02–1.10	0.002	rectal	1.21	1.05–1.38	0.007	1.23	1.07–1.41	0.003	rectal	0.94	0.72–1.24	0.667			

CA: Classical adenocarcinoma; MA: Mucinous adenocarcinoma; SRCC: Signet-ring cell carcinoma; CEA: Carcinoembryonic antigen; C0: CEA-normal; C1: CEA-elevated; OS: Overall survival; CSS: Cancer-specific survival; CI: confidence interval; CRC: Colorectal cancer.

To further investigate the association between serum CEA levels (C0 and C1) and histopathologic type in determining the prognosis and metastasis status of CRC patients, we defined an interaction variable called Histopathologic type and serum CEA level (H&CEA). This new variable was included in the Cox regression analysis to separately predict OS or CSS in CRC patients. As shown in Table 3, H&CEA was an independent prognostic factor (*P* < 0.001). When CEA levels were normal, CA patients exhibited a lower risk than both MA and SRCC patients. However, in the context of elevated serum CEA levels, CA patients presented a significantly higher risk of OS mortality than patients with the other two histopathologic subtypes. In CRC patients with CA, an elevated serum CEA level was associated with a 70.0% increased risk of mortality compared to those with normal serum CEA levels. These results are consistent with the study of CSS mortality (78.0%) (Table 3).

**Table 3:** Univariate and multivariate analyses of OS and CSS in CRC patients

**Variables**	**Univariate analysis of OS**			**Multivariate analysis of OS**			**Univariate analysis of CSS**			**Multivariate analysis of CSS**		
	**Hazard ratio**	**95% CI**	***P***	**Hazard ratio**	**95% CI**	***P***	**Hazard ratio**	**95% CI**	***P***	**Hazard ratio**	**95% CI**	***P***
*Race*												
White	Ref			Ref			Ref			Ref		
Black	1.35	1.30–1.41	<0.001	1.34	1.29–1.39	<0.001	1.43	1.37–1.5	<0.001	1.35	1.29–1.42	<0.001
Other	0.89	0.86–0.93	<0.001	0.95	0.91–0.99	0.016	0.96	0.91–1.01	0.091	1.01	0.96–1.07	0.573
*Sex*												
Male	Ref			Ref			Ref			Ref		
Female	0.88	0.85–0.90	<0.001	0.82	0.80–0.84	<0.001	0.93	0.9–0.96	<0.001	0.89	0.86–0.92	<0.001
*Age*												
<=65	Ref			Ref			Ref					
>65	1.58	1.54–1.63	<0.001	1.93	1.88–1.99	<0.001	1.11	1.08–1.15	<0.001	1.49	1.44–1.54	<0.001
*Grade*												
I	Ref			Ref			Ref			Ref		
II	1.18	1.12–1.25	<0.001	0.98	0.92–1.04	0.446	1.44	1.33–1.55	<0.001	1.05	0.97–1.13	0.225
III	1.91	1.79–2.03	<0.001	1.23	1.16–1.31	<0.001	2.68	2.47–2.91	<0.001	1.41	1.3–1.53	<0.001
IV	1.99	1.81–2.18	<0.001	1.25	1.14–1.38	<0.001	2.71	2.42–3.05	<0.001	1.4	1.25–1.58	<0.001
*Stage*												
Distant	Ref			Ref			Ref			Ref		
Localized	0.14	0.14–0.15	<0.001	0.62	0.54–0.70	<0.001	0.06	0.06–0.06	<0.001	0.37	0.32–0.43	<0.001
Regional	0.22	0.21–0.23	<0.001	0.73	0.65–0.82	<0.001	0.17	0.16–0.17	<0.001	0.68	0.59–0.77	<0.001
*T*												
T1	Ref			Ref			Ref			Ref		
T2	0.86	0.81–0.91	<0.001	0.83	0.78–0.88	<0.001	0.66	0.61–0.73	<0.001	0.64	0.58–0.7	<0.001
T3	1.5	1.43–1.57	<0.001	0.95	0.91–1.00	0.046	1.9	1.79–2.02	<0.001	0.87	0.81–0.93	<0.001
T4	3.25	3.09–3.42	<0.001	1.37	1.29–1.45	<0.001	5	4.69–5.33	<0.001	1.34	1.24–1.44	<0.001
*N*												
N0	Ref			Ref			Ref					
N1	1.54	1.49–1.59	<0.001	1.09	1.05–1.13	<0.001	2.29	2.2–2.39	<0.001	1.2	1.15–1.26	<0.001
N2	2.83	2.74–2.92	<0.001	1.49	1.43–1.55	<0.001	4.73	4.55–4.93	<0.001	1.73	1.65–1.82	<0.001
*M*												
M0	Ref			Ref			Ref			Ref		
M1	5.68	5.52–5.85	<0.001	2.85	2.54–3.21	<0.001	8.82	8.52–9.12	<0.001	3.26	2.86–3.73	<0.001
*Site*												
Right	Ref			Ref			Ref			Ref		
Left	0.88	0.86–0.91	<0.001	0.89	0.87–0.92	<0.001	0.96	0.92–0.99	0.024	0.9	0.87–0.94	<0.001
rectal	0.91	0.88–0.94	<0.001	1.08	1.04–1.12	<0.001	1.03	0.99–1.08	0.116	1.19	1.14–1.25	<0.001
*H&CEA*												
CA&C0	Ref			Ref			Ref			Ref		
CA&C1	2.64	2.56–2.71	<0.001	1.7	1.65–1.75	<0.001	3.5	3.37–3.62	<0.001	1.78	1.72–1.85	<0.001
MA&C0	1.33	1.24–1.44	<0.001	1.16	1.07–1.24	<0.001	1.46	1.33–1.61	<0.001	1.2	1.09–1.32	<0.001
MA&C1	2.52	2.36–2.68	<0.001	1.55	1.46–1.66	<0.001	3.32	3.08–3.58	<0.001	1.69	1.56–1.82	<0.001
SRCC&C0	3.22	2.73–3.8	<0.001	1.85	1.56–2.19	<0.001	4.57	3.80–5.50	<0.001	1.96	1.62–2.36	<0.001
SRCC&C1	4.7	4.09–5.41	<0.001	2.49	2.16–2.87	<0.001	6.78	5.81–7.91	<0.001	2.72	2.32–3.18	<0.001

CA: Classical adenocarcinoma; MA: Mucinous adenocarcinoma; SRCC: Signet-ring cell carcinoma; CEA: Carcinoembryonic antigen; C0: CEA-normal; C1: CEA-elevated; OS: Overall survival; CSS: Cancer-specific survival; CI: Confidence interval; H&CEA: Histopathologic type and serum CEA level; CRC: Colorectal cancer.

### Effect of CEA on the prognosis of patients with different histopathologic types of CRC

**Figure 2.**
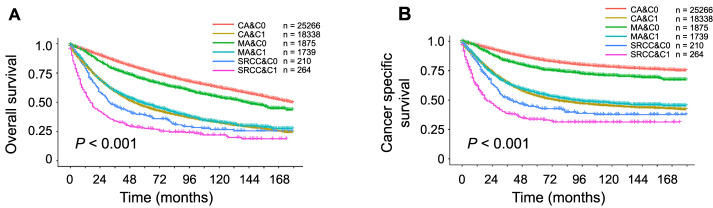
**Kaplan–Meier survival curves for patients with different histopathological types and serum CEA level.** (A) OS; (B) CSS. OS: Overall survival; CEA: Carcinoembryonic antigen; CSS: Cancer-specific survival.

K–M survival curves are plotted in Figure 2. The 3-year OS rates were 85.42% for CA&C0, 59.05% for CA&C1, 77.99% for MA&C0, 59.49% for MA&C1, 46.71% for SRCC&C0, and 34.07% for SRCC&C1 (Figure 2A, *P* < 0.001). Therefore, CA&C1 patients presented a 3-year OS rate similar to that of MA&C1 patients (59.05 vs 59.49%, *P* ═ 0.703). Although the prognosis of CRC patients was worsened by elevated serum CEA levels in all three histopathologic types, the CA group was the most affected (59.05% vs 85.42%, *P* < 0.001). In addition, these findings are consistent with the results for CSS. As shown in Figure 2B, the 3-year CSS rates were 83.92% for CA&C0, 64.56% for CA&C1, 83.25% for MA&C0, 64.15% for MA&C1, 51.28% for SRCC&C0, and 35.42% for SRCC&C1 (*P* < 0.001). CA&C1 presented a significantly lower 3-year CSS rate than did CA&C0 (64.56 vs 83.52%, *P* < 0.001) but presented a similar rate to that of MA&C1 (64.56 vs 64.15%, *P* ═ 0.748).

**Figure 3.**
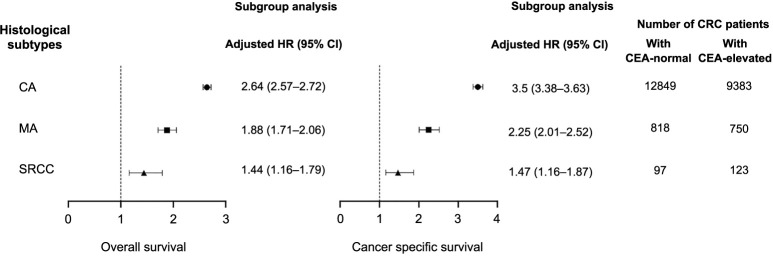
**Subgroup analysis.** Survival comparison between patient groups with normal and elevated preoperative serum CEA levels. CEA: Carcinoembryonic antigen.

**Table 4:** Distribution of metastasis in patients with different histopathologic types

**Classical adenocarcinoma (CA)**				**Mucinous adenocarcinoma (MA)**				**Signet-ring cell carcinoma (SRCC)**			
**Variables**	**CEA-normal**	**CEA-elevated**	***P***	**Variables**	**CEA-normal**	**CEA-elevated**	***P***	**Variables**	**CEA-normal**	**CEA-elevated**	***P***
	***n* ═ 12,849** ***n*(%)**	***n* ═ 9383** ***n*(%)**			***n* ═ 818** ***n*(%)**	***n* ═ 750** ***n*(%)**			***n* ═ 97** ***n*(%)**	***n* ═ 123** ***n*(%)**	
*Peritoneum*				*Peritoneum*				*Peritoneum*			
Yes	194 (1.51)	844 (8.99)		Yes	36 (4.4)	86 (11.47)		Yes	18 (18.56)	27 (21.95)	
No	12655 (98.49)	8539 (91.01)	<0.001	No	782 (95.6)	664 (88.53)	<0.001	No	79 (81.44)	96 (78.05)	0.615
*Liver*				*Liver*				*Liver*			
Yes	508 (3.95)	2386 (25.43)		Yes	31 (3.79)	96 (12.8)		Yes	3 (3.09)	11 (8.94)	
No	12341 (96.05)	6997 (74.57)	<0.001	No	787 (96.21)	654 (87.2)	<0.001	No	94 (96.91)	112 (91.06)	0.098
*Distant lymph node*				*Distant lymph node*				*Distant lymph node/Bone/Brain/Lung*			
Yes	153 (1.19)	438 (4.67)		Yes	7 (0.86)	22 (2.93)		Yes	4 (1.03)	23 (4.67)	
No	12696 (98.81)	8945 (95.33)	<0.001	No	811 (99.14)	728 (97.07)	0.002	No	384 (98.97)	469 (95.33)	0.001
*Bone*				*Bone/Brain/Lung*							
Yes	18 (0.14)	122 (1.3)		Yes	9 (0.37)	30 (1.33)					
No	12831 (99.86)	9261 (98.7)	<0.001	No	2445 (99.63)	2220 (98.7)	<0.001				
*Brain*											
Yes	10 (0.08)	35 (0.37)									
No	12839 (99.92)	9348 (99.63)	<0.001								
*Lung*											
Yes	147 (1.14)	702 (7.48)									
No	12702 (98.86)	8681 (92.52)	<0.001								

CEA: Carcinoembryonic antigen.

Furthermore, OS and CSS were compared between the normal- and elevated-CEA groups for patients with different histopathologic subtypes using the HRs in the forest plot (Figure 3). In terms of OS, compared to the normal-level group, elevated serum CEA was associated with an 88% increased risk of OS in MA patients (HR ═ 1.88, 95% confidence interval (CI) ═ 1.71–2.06, *P* < 0.001); in SRCC patients, elevated serum CEA was associated with a 44% increased risk of OS (HR ═ 1.44, 95% CI ═ 1.16–1.79, *P* < 0.001); however, in CA patients, elevated serum CEA was associated with an even greater risk of OS (HR ═ 2.64, 95% CI ═ 2.57–2.72, *P* < 0.001).

These findings were even more pronounced for CSS. Compared to those with normal CEA levels, abnormally high serum CEA levels were linked to a 125% increased risk of cancer-specific mortality in MA patients (HR ═ 2.25, 95% CI ═ 2.01–2.52, *P* < 0.001) and a 47% increased risk in SRCC patients. In contrast, the increased risk of cancer-specific mortality in adenocarcinoma patients was significantly greater than in the other two groups, at about 250% (HR ═ 3.5, 95% CI ═ 3.38–3.63, *P* < 0.001).

### Significance of the serum CEA concentration for the risk of liver metastasis in patients with various histopathologic types

Expert consensus has shown that the liver is the most common site of metastasis for CRC [10]. However, the specific factors affecting the incidence of liver metastasis in patients with different pathological types have not been studied in detail. To explore the synergistic effect of CEA and histological type on liver metastases, we first performed a chi-square analysis on patients in different pathological subgroups with various metastases, as shown in Table 4. Due to the small size or lack of some subgroups analyzed, and to present the group proportion data as fully as possible, we grouped the metastatic sites in these three pathological type groups in different ways. In the CA cohort, peritoneum, liver, distant lymph nodes, bone, brain, and lung metastases were included; in the MA cohort, bone, brain, and lung metastases were combined; and in contrast, the other four metastases, excluding peritoneum and liver, were combined as one factor for discussion in the SRCC cohort.

The results showed that patients with CA had significantly more liver metastases than patients with MA or SRCC. Furthermore, in SRCC, elevated CEA had no significant effect on either peritoneal metastasis (*P* ═ 0.615) or liver metastasis (*P* ═ 0.098). In MA, although elevated CEA levels significantly increased the incidence of both peritoneal metastasis (*P* < 0.001) and liver metastasis (*P* < 0.001), the incidence of the two metastases was similar when CEA levels were normal. Similarly, the incidence of metastases with normal CEA levels was similar (4.40% vs 3.79%, *P* ═ 0.533), and no significant differences were observed in the group with elevated CEA levels (11.47% vs 12.80%, *P* ═ 0.429).

Based on the above results, our attention shifted from the effect of CEA on the incidence of metastases at different sites in MA and SRCC. However, unlike the above results, we observed a specific role for CEA in the development of liver metastasis in CA. High preoperative serum CEA levels increased the proportion of liver metastases in CA (25.43% vs 3.95%, *P* < 0.001), while the incidence of peritoneal metastases increased by only 7.48% (8.99% vs 1.51%, *P* < 0.001). In conclusion, elevated serum CEA levels were associated with a high incidence of liver metastasis in patients with adenocarcinoma.

In Table 4, we only discuss whether metastasis occurred and ignore the effect of multisite metastasis. To verify whether the specific effect of CEA on liver metastasis is influenced by combined multisite metastasis, we further performed a chi-square analysis of single-site and multiple-site metastasis in CA patients. As shown in Table 5, after excluding interference from multiple-site metastasis, we still observed significant differences in the distribution of liver metastases (19.38% vs 3.34%, *P* < 0.001), which further confirmed the previous conclusion.

**Table 5:** Distribution of single-or multiple-site metastasis in patients of CA

**Variables**	**Classical adenocarcinoma (CA)**		
	**CEA-normal**	**CEA-elevated**	***P***
	***n* ═ 12849** ***n*(%)**	***n* ═ 9383** ***n*(%)**	
No met	12012 (93.49)	6338 (67.55)	
Peritoneum	99 (0.77)	175 (1.87)	
Distant lymph node	82 (0.64)	135 (1.44)	
Bone	5 (0.04)	19 (0.2)	
Brain	8 (0.06)	11 (0.12)	
Liver	430 (3.35)	1818 (19.38)	
Lung	75 (0.58)	172 (1.83)	
Multi	138 (1.07)	715 (7.62)	<0.001

CEA: Carcinoembryonic antigen; CA: Classical adenocarcinoma.

**Figure 4.**
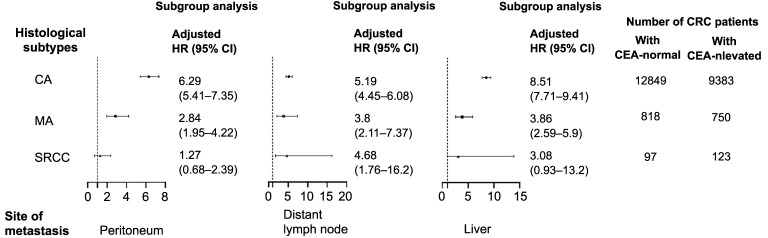
Comparison of single-site metastasis development between the C0 and C1 groups across respective histopathological types.

Finally, logistic regression analysis was performed to determine the HRs to compare the development of single-site metastasis between the normal- and elevated-CEA groups according to the respective histopathologic type. Images of peritoneal metastasis, distant lymphatic metastasis, and liver metastasis are presented in Figure 4. Compared with normal serum CEA levels, elevated serum CEA levels were associated with a 529% increased risk of peritoneal metastasis (HR ═ 6.29, 95% CI ═ 5.41–7.35; *P* < 0.001), a 419% increase in distant lymphatic metastasis (HR ═ 5.19, 95% CI ═ 4.45–6.08; *P* < 0.001) and even a 751% increase in liver metastasis (HR ═ 8.51, 95% CI ═ 7.71–9.41; *P* < 0.001).

## Discussion

Our pioneering, large-scale population study has, for the first time, verified that preoperative serum CEA levels, coupled with CRC histological subtypes, alter prognosis and metastasis risks. While MA generally presents more favorably, we’ve pinpointed a subset of CRC patients with elevated preoperative CEA levels who exhibit notably worse outcomes. Furthermore, the novel H&CEA interactive variable emerges as an independent prognostic indicator for CRC.

CEA, a nonspecific serum biomarker, is elevated in multiple malignancies, including gastric, thyroid, breast, ovarian, and lung cancers [11–13]. In CRC, it is vital for efficacy assessment [14]. Focusing on adenocarcinoma, the predominant CRC type (>90% by AJCC), we subdivided it into CA, MA, and SRCC. However, few studies have compared CEA’s impact across these subtypes. Thus, we analyzed a large cohort to explore the relationship between preoperative CEA levels, histological subtypes, and CRC prognosis/metastasis.

Among the 47,692 CRC patients in the 2004–2015 SEER database, 42.65% (20,341) exhibited elevated preoperative CEA levels. Elderly (>80) patients face surgical challenges due to compromised functions and comorbidities like hypertension, CHD, and diabetes [15, 16], heightening postoperative complication risks. This underscores the need for individualized surgical assessments. Focusing on ≤79-year-olds [17–19], we found significant variations in race, sex, differentiation, TNM stage, tumor location, and histology, but not age. Notably, black women, MA/SRCC patients, those with large right-sided/rectal tumors, poor differentiation, or distant metastasis were more prone to elevated CEA. This finding also confirmed the high degree of malignancy in patients with high CEA expression.

Previous studies have established a link between CRC histological subtypes and patient prognosis, with MA patients experiencing significantly poorer outcomes than CA patients, and SRCC serving as an independent poor prognosis indicator [5, 6, 20]. Cox regression analysis identified race, sex, age, differentiation, TNM stage, CEA level, tumor location, and histopathological type as key prognostic factors. Notably, older age (>65), advanced stages (T3-T4, N1-N2, M1), high CEA expression, and MA/SRCC classification emerged as primary risk factors, while female gender and left colon/rectum tumors were protective, consistent with previous studies [21]. Surprisingly, despite its prognostic risk, SRCC patients fared better than MA but worse than CA. However, the poor prognosis of some nonmucinous CRCs highlights the need to understand shared features among poor prognosis CAs. We hypothesized that preoperative CEA levels could reveal the poor prognosis of specific pathological types. Subsequent subgroup analysis confirmed elevated CEA as an independent risk factor across all subtypes, but CA showed a significantly higher risk ratio. To further validate our hypothesis, we introduced an interaction variable (H&CEA). Our analysis found that C0 combined with CA had lower risk values compared to C0 with MA or SRCC. However, in the C1 subgroup, the OS and CSS risks in the CA subgroup surpassed those of the other subtypes, approaching those of MA with C1. Kaplan–Meier survival curves and forest plots reinforced our findings, revealing the most pronounced decline in CRC CA prognosis with elevated CEA. In conclusion, the H&CEA interaction variable emerges as an independent prognostic factor in CRC, with implications for CRC diagnosis and personalized treatment strategies.

In addition to prognosis, another key feature of tumors is the occurrence of metastasis. Many CRC patients have nonsignificant and atypical early symptoms, and by the time obvious clinical symptoms appear, the cancer is already in the middle or late stages, often with metastasis. This can be fatal, leading to space-occupying effects and internal environment imbalances, seriously affecting patient health and quality of life. In past decades, approximately 20%–25% of patients with an initial CRC diagnosis had metastasis [22]. In terms of pathophysiological mechanisms, CEA promotes cell adhesion, proliferation, and migration *in vitro* and *in vivo*, driving the adhesion of colon cancer cells to metastatic sites, and forming metastatic tumor foci [23]. A systematic study showed that different metastatic sites have varying effects on patient prognosis, and the ability to predict metastatic sites holds significant importance [24]. The liver is the most common site of CRC metastasis and the leading cause of death. The mechanism of liver metastasis can be explained by CEA binding to hnRNP M on Kupffer cells, leading to the activation and production of proinflammatory and anti-inflammatory cytokines. These cytokines influence the upregulation of adhesion molecules on the hepatic sinusoidal endothelium, controlling tumor cell engraftment and survival in the liver [25]. Recent studies have further identified the mechanism by which CEA promotes liver metastasis, with Ma et al. [25] showing that CEA expression is upregulated by the activation of the c-KIT-ERK 1/2 signaling pathway, promoting CRC progression.

Our dataset analysis indicates that liver metastasis is more concentrated in CA, with a liver metastasis rate of 13.00%, much greater than the rates of lymphatic or lung metastasis. However, MA and SRCC patients had a higher incidence of peritoneal metastasis, consistent with the conclusions of a national retrospective study by Hugen et al. [26].

Importantly, our analysis of the distribution of metastatic sites in patients with different histological subtypes revealed that high preoperative serum CEA levels increased the proportion of liver metastasis in CA by 21.48% and peritoneal metastasis by only 7.48%. High CEA expression in MA and SRCC increased liver metastases by only 9.01% and 5.89%, respectively. After excluding interference from multisite metastasis, we still observed a significant difference in liver metastasis distribution (19.38% vs 3.34%, *P* < 0.001). Therefore, we concluded that elevated serum CEA levels were associated with a high incidence of liver metastasis in CA. This phenomenon may be strongly linked to the pathophysiological mechanisms of CEA. One year after surgery, CEA’s cell adhesion and metastasis functions are promoted, leading to a high incidence of tumor metastasis. Additionally, since the liver is the primary metabolic organ for CEA, liver dysfunction may cause false positives due to elevated CEA levels. Evidence also suggests that CEA concentration is the most sensitive test for detecting liver metastases in CRC patients eligible for surgical removal. Patients with colon cancer liver metastases and a preoperative CEA concentration ≤30 µg/L are more likely to have resectable metastases, thereby improving survival [27, 28]. This study provides theoretical support for the clinical diagnosis and treatment of liver metastasis in CRC patients and its prevention.

The key strength of this study is that it is the first comprehensive exploration of the relationship between histological classification and preoperative serum CEA levels in determining CRC prognosis and metastasis. We defined a new interaction variable by combining histological classification and CEA concentration, which is clinically significant, low cost, and easy to use for diagnosis. More importantly, we identified a subgroup of patients with colorectal CA with a very poor prognosis compared to other CA patients, suggesting that elevated preoperative serum CEA in colorectal CA should receive greater attention from oncologists.

However, there are several limitations to this study. First, it was a retrospective study, which introduces potential bias. Second, the SEER database is large and susceptible to coding errors and censored data. The long period covered by the samples may have impacted the consistency of CEA judgment. Third, liver damage can reduce the liver’s uptake and degradation of circulating CEA, potentially increasing serum CEA levels [29, 30], and creating bias in patients with liver dysfunction. Additionally, CEA levels are influenced by various other factors, including the presence of other tumors, pneumonia, cardiovascular diseases, diabetes, specific diets, and the use of certain hormones and antibiotics. However, due to the inherent limitations of public databases, we were unable to analyze all factors contributing to CEA level variations or track individual patients’ serial CEA changes. Consequently, we focused on the impact of relative preoperative CEA levels in a large, data-driven population on prognosis. Unfortunately, the limitations of public data prevented us from discussing other key variables. Moreover, the SEER database lacks information on genetic mutation status, oral medications, and surgery, which may have affected the results.

Despite these limitations, this study advances our understanding of CRC prognosis and metastasis. Future prospective studies are needed to explore the effects of surgery, radiotherapy, and other tumor markers on CRC prognosis and liver metastasis. Additionally, the molecular mechanisms of CEA in CRC metastasis and malignancy remain unknown, necessitating further research.

## Conclusion

In conclusion, our study confirms the importance of the histological subtype in determining the prognosis of CRC patients with elevated CEA levels. The newly defined interaction variable H&CEA may serve as an independent prognostic factor for CRC and be included in the diagnosis of CRC as well as in the determination of individual treatment options. Among all groups categorized by histological subtype and CEA level, patients with elevated CEA levels deserve special attention due to their prognosis and higher risk of liver metastasis. This study broadens our understanding of the interactive influence of histological heterogeneity and preoperative serum CEA levels on patient outcomes, which are associated with pathophysiological mechanisms, and may help in monitoring and predicting the prognosis of CEA-elevated CA patients.

## Supplemental data

**Table S1:** Univariate and multivariate analyses for CRC patients

**Variables**	**Univariate analysis of OS**			**Multivariate analysis of OS**		
	**Hazard** **ratio**	**95% CI**	***P***	**Hazard** **ratio**	**95% CI**	***P***
**Race**						
White	Ref			Ref		
Black	1.35	1.30–1.41	<0.001	1.34	1.29–1.40	<0.001
Other	0.89	0.86–0.93	<0.001	0.95	0.91–0.99	0.017
**Sex**						
Male	Ref			Ref		
Female	0.88	0.85–0.90	<0.001	0.82	0.80–0.84	<0.001
**Age**						
<=65	Ref			Ref		
>65	1.58	1.54–1.63	<0.001	1.93	1.88–1.99	<0.001
**Grade**						
I	Ref			Ref		
II	1.18	1.12–1.25	<0.001	0.98	0.93–1.04	0.501
III	1.91	1.79–2.03	<0.001	1.23	1.16–1.31	<0.001
IV	1.99	1.81–2.18	<0.001	1.25	1.14–1.38	<0.001
**Stage**						
Distant	Ref			Ref		
Localized	0.14	0.14–0.15	<0.001	0.61	0.54–0.69	<0.001
Regional	0.22	0.21–0.23	<0.001	0.73	0.65–0.82	<0.001
**T**						
T1	Ref			Ref		
T2	0.86	0.81–0.91	<0.001	0.83	0.78–0.88	<0.001
T3	1.5	1.43–1.57	<0.001	0.95	0.91–1.00	0.057
T4	3.25	3.09–3.42	<0.001	1.37	1.29–1.45	<0.001
**N**						
N0	Ref			Ref		
N1	1.54	1.49–1.59	<0.001	1.09	1.05–1.13	<0.001
N2	2.83	2.74–2.92	<0.001	1.49	1.43–1.55	<0.001
**M**						
M0	Ref			Ref		
M1	5.68	5.52–5.85	<0.001	2.86	2.54–3.21	<0.001
**CEA**						
C0	Ref			Ref		
C1	2.56	2.49–2.63	<0.001	1.66	1.61–1.71	<0.001
**Site**						
Right	Ref			Ref		
Left	0.88	0.86–0.91	<0.001	0.89	0.87–0.92	<0.001
Rectal	0.91	0.88–0.94	<0.001	1.08	1.04–1.12	<0.001
**Histopathologic type**						
CA	Ref			Ref		
MA	1.17	1.12–1.23	<0.001	1	0.96–1.05	0.843
SRCC	2.51	2.26–2.79	<0.001	1.6	1.44–1.79	<0.001

CEA: Carcinoembryonic antigen; C0: CEA-normal; C1: CEA-elevated; CA: Classical adenocarcinoma; MA: Mucinous adenocarcinoma; SRCC: Signet-ring cell carcinoma; OS: Overall survival.

## Data Availability

The data in this study were collected from an open public database and can be accessed through https://seer.cancer.gov/.
